# Influences on surgical antimicrobial prophylaxis decision making by surgical craft groups, anaesthetists, pharmacists and nurses in public and private hospitals

**DOI:** 10.1371/journal.pone.0225011

**Published:** 2019-11-14

**Authors:** Courtney Ierano, Karin Thursky, Trisha Peel, Arjun Rajkhowa, Caroline Marshall, Darshini Ayton

**Affiliations:** 1 National Health and Medical Research Council Centre of Research Excellence: National Centre for Antimicrobial Stewardship (NCAS), Peter Doherty Research Institute for Infection and Immunity Melbourne, Victoria, Australia; 2 Department of Medicine, Royal Melbourne Hospital, Faculty of Medicine, Dentistry and Health Sciences, University of Melbourne, Parkville, Victoria, Australia; 3 Department of Infectious Diseases, Peter MacCallum Cancer Centre, Melbourne, Victoria, Australia; 4 Department of Infectious Diseases, Faculty of Medicine, Nursing and Health Sciences, Alfred Health/Monash University, Melbourne, Victoria, Australia; 5 Infection Prevention and Surveillance Service, Royal Melbourne Hospital, Parkville, Victoria, Australia; 6 Victorian Infectious Diseases Service (VIDS), Royal Melbourne Hospital, Parkville, Victoria, Australia; 7 Department of Epidemiology and Preventive Medicine, Faculty of Medicine, Nursing and Health Sciences, Monash University, Melbourne, Victoria, Australia; Federal University of Sergipe, BRAZIL

## Abstract

**Background:**

Surgical antimicrobial prophylaxis (SAP) is a leading indication for antibiotic use in the hospital setting, with demonstrated high rates of inappropriateness. Decision-making for SAP is complex and multifactorial. A greater understanding of these factors is needed to inform the design of targeted antimicrobial stewardship interventions and strategies to support the optimization of SAP and its impacts on patient care.

**Methods:**

A qualitative case study exploring the phenomenon of SAP decision-making. Focus groups were conducted with surgeons, anaesthetists, theatre nurses and pharmacists across one private and two public hospitals in Australia. Thematic analysis was guided by the Theoretical Domains Framework (TDF) and the Capabilities, Opportunities, Motivators-Behaviour (COM-B) model.

**Results:**

Fourteen focus groups and one paired interview were completed. Ten of the fourteen TDF domains were identified as relevant. Thematic analysis revealed six significant themes mapped to the COM-B model, and subthemes mapped to the relevant TDF domains in a combined framework. Key themes identified were: 1) Low priority for surgical antimicrobial prophylaxis prescribing skills; 2) Prescriber autonomy takes precedence over guideline compliance; 3) Social codes of prescribing reinforce established practices; 4) Need for improved communication, documentation and collection of data for action; 5) Fears and perceptions of risk hinder appropriate SAP prescribing; and 6) Lack of clarity regarding roles and accountability.

**Conclusions:**

SAP prescribing is a complex process that involves multiple professions across the pre-, intra- and post-operative surgical settings. The utilisation of behaviour change frameworks to identify barriers and enablers to optimal SAP prescribing supports future development of theory-informed antimicrobial stewardship interventions. Interventions should aim to increase surgeon engagement, enhance the prioritisation of and accountability for SAP, and address the underlying social factors involved in SAP decision-making, such as professional hierarchy and varied perceptions or risks and fears.

## Introduction

Surgical antibiotic prophylaxis (SAP) involves the administration of antibiotics for the purpose of preventing surgical site infections (SSIs), and is a well-established infection prevention measure. As such, SAP is a leading indication for antibiotic use in the hospital setting[1, 2].

Australian and international studies have observed high rates of inappropriate SAP prescribing[1–9], which may consequently contribute to sub-optimal patient care and outcomes, and is also considered a driver of the emergence of antimicrobial resistance (AMR) [10]. Thus, appropriate use of SAP is desired to improve patient care and reduce the burden of inappropriate prescribing on both the patient and health care system.

Antimicrobial stewardship (AMS) has been identified in global policy initiatives[11, 12] as a key clinical strategy for the containment of AMR. It responds to the emergence of AMR by aiming to reduce inappropriate use of antimicrobial medications and thus conserve the effectiveness of these medications; its primary aim is the enhancement of patient care through improved management of antimicrobial therapy [13]. AMS programs and interventions vary immensely. The development and tailoring of AMS interventions to target areas of high concern, such as surgical prophylaxis, is warranted to further optimise antimicrobial usage and patient health.

This study examines clinicians’ perceptions across public and private hospitals as research demonstrates a difference in SAP guideline compliance between the two settings [14]. Public hospitals generally deal with more complicated and acute cases, while private hospitals have higher rates of elective and day procedures [14, 15]. This difference may contribute to observed variations in SAP practices.

This qualitative study utilises the Theoretical Domains Framework (TDF)[16] and COM-B model[17, 18] to explore and describe decision-making processes for SAP prescribing. It is important to understand the underlying factors that drive SAP prescribing. AMS interventions aimed at enhancing the appropriateness of SAP prescribing need to consider the contextual and environmental factors that influence SAP prescribing. The utilisation of theoretical frameworks for the identification of barriers and enablers to prescribing can assist in the development of AMS interventions that may successfully be implemented and sustained [19].

## Methods

### Aim

The primary aim of this study was to identify barriers and enablers of appropriate SAP prescribing and evidence-based guideline compliance. The secondary aim was to compare the perceptions of health professionals in surgical specialties across both public and private hospital settings regarding these barriers and enablers.

### Project type and design

Exploratory, multi-site, collective qualitative case study. Exploring the phenomenon of decision-making processes related to SAP prescribing and guideline compliance across three tertiary public and private hospitals in Australia.

### Participant recruitment

Recruitment was iterative and aimed at achieving adequate representation from key stakeholders and qualitative data saturation. Surgeons, anaesthetists, pharmacists and theatre nurses were identified via purposive sampling, which was complemented with a snowball sampling strategy.

Conducting sixteen focus group discussions, with a minimum of four participants in each, was proposed to gain knowledge of decision-making for SAP prescribing from multiple perspectives across a range of surgical specialties. Surgical specialties including orthopaedics, general surgery and cardiac surgery were targeted as they represent the most commonly performed procedures in Australia [15]. Additional specialties such as vascular, and plastic and reconstructive surgery were targeted to ensure representation of surgical specialties with demonstrated high rates of inappropriate SAP, as informed by the Australian Surgical National Antimicrobial Prescribing Survey (Surgical NAPS)[4]. Focus groups targeted these specialty groups separately.

In circumstances where four participants were recruited, but fewer than four attended, the focus group still proceeded. In circumstances where four participants could not be recruited, a semi-structured paired interview was conducted instead.

Project Information Statement letters were sent to the key informant and/or organiser of the focus group and distributed to potential participants via email. Additional copies of the Project Information Statement letter were available at the focus group meeting, to ensure all participants were aware of the details of the project and to facilitate provision of consent. Implied consent was provided when participants attended the focus group.

This research project was granted ethical approval following low-risk review the Alfred Hospital Human Research Ethics Committee (HREC) on the 11/10/2017 and the Epworth HealthCare HREC on the 30/10/2017. Local governance approval was sought from both Alfred Health and Epworth HealthCare in addition to Melbourne Health (Ethics approval inclusive of Alfred Health HREC approval). Ethics approval included consent for participation and potential publication.

### Data collection

Focus group guides based upon the TDF[16] and COM-B [18] were developed, and adapted for the speciality staff participating. Questions pertained to roles and responsibilities for SAP prescribing, factors that influence SAP decision-making, and strategies and resources to facilitate change (Tables A and B in S1 Appendix). Focus groups were audio-recorded and transcribed.

### Data analysis

The phases of thematic analysis[20] were adapted to reflect the utilisation of the TDF (a behaviour change framework) for the codebook structure and deductive coding. Theme codes were developed for each of the 14 domains within the TDF. Deductive and inductive coding was based upon these theme codes.

Focus group transcripts were the primary data source and were imported into the data management software NVivo11®. Coding for thematic analysis was undertaken by researcher CI. Approximately 20% of transcripts was double-coded by researcher AR. The coding strategy was reviewed iteratively by CI, AR and DA to promote rigour.

Deductive coding identified key TDF domains[16], which were mapped to the COM-B model [18, 21]. Concurrent inductive coding enabled identification of emerging themes; where possible, inductive codes were matched to, or sub categorised within the fourteen TDF theme nodes as sub-codes. Relevant and dominant themes were identified through consensus between two researchers (CI and AR).

## Results

Fourteen focus groups and one paired interview were completed. Table 1 summarises the characteristics of the focus groups/interview and their participants. The average number of participants was five and the average duration 47 minutes.

**Table 1:** Focus group participant details.

Focus group code	Site Funding	Health Profession	Participants (n)	Duration^ (mins)
F1.1TN	Public	Theatre Nurses	8	32
F2.1A	Public	Anaesthetists	3*	56
F3.2A	Private	Anaesthetists	3*	64
F4.3CS	Public	Surgeons- colorectal	9	54
F5.3PS	Public	Surgeons- plastic and reconstructive	4	56
F6.3NS	Public	Surgeons- neurological	4	58
F7.2VS	Private	Surgeons- vascular	2	28
F8.3US	Public	Surgeons- urological	3*	39
F9.1CTS	Public	Surgeons- cardiothoracic	13	22
F10.3A	Public	Anaesthetists	4	64
F11.2TN	Private	Theatre Nurses	5	49
F12.1P	Public	Pharmacists	4	42
F13.2P	Private	Pharmacists	4	55
F14.3SR	Public	Surgical registrars and residents	6	46
F15.3OS	Public	Surgeons- orthopaedics	5	42

^ Seconds rounded off to the nearest minute

* One of the four participants became unavailable at the last minute.

The analysis of SAP prescribing behaviour revealed six significant themes with additional sub-themes. These themes were mapped to the relevant TDF and COM-B domains (Table C in S2 Appendix) and are described in detail below.

### Theme 1. Low priority for surgical antimicrobial prophylaxis prescribing skills

Most participants, across the specialties, deemed SAP prescribing skills to be a low priority, and characterised surgical site infections and post-operative complications as *“complex issues”* (F9, public cardiothoracic surgeon) that can’t be solely prevented by the administration of SAP. Participants noted that multiple factors contribute to the risk of infection, and that *“you can’t just draw it down to antibiotics*, *hand washing*, *or whether there are cobwebs in the ceiling in the operating theatre*” (F9, public cardiothoracic surgeon).

#### 1.1: Surgical technique is of greater importance

Participating surgeons highlighted that, in terms of infection prevention and control, greater importance was placed on surgical technique than on how antibiotics are prescribed in the surgical setting (Table 2). This was, therefore, considered a barrier to the optimisation of SAP prescribing.

**Table 2:** Representative quotations from Theme 1 reflecting ‘Physical Capability’ of the COM-B model.

Theme 1: Low priority for surgical antimicrobial prophylaxis prescribing skills.			
Subtheme	TDF Domain	Illustrative quotes	Barrier or Enabler?
Surgical technique of greater importance	Skills	*“The thing that’s going to reduce the infection rates is technique*.*”* (F6, public neurosurgeon)	Barrier
		*“I don’t think that giving them more antibiotics makes the infections less likely… a lot of it has to do with my technique… I don’t feel that I can overcome my failings that much with antibiotics*.*”* (F4, public colorectal surgeon)	
Deskilling surgeons		*“We were discouraged from [prescribing antibiotics] and discouraged from doing the fluids because we were taking it away from the residents*. *Yeah*, *de-skilling the residents*.*”* (F10, public anaesthetist)	Barrier

#### 1.2: Deskilling surgeons

The need to improve SAP prescribing was identified, but not at the expense of surgical technique. When asked about the inclusion of other professionals’ involvement in SAP prescribing (i.e., AMS, infectious diseases [ID] and anaesthetists), surgical participants expressed apprehensions about the potential deskilling of surgeons (Table 2) and reinforced the importance of surgeons maintaining their prescribing skills and autonomy.

### Theme 2. Prescriber autonomy takes precedence over guideline compliance

Prescriber autonomy and discretion were considered to be paramount when participants described how SAP decisions were made. Participants were notably aware of guidelines; however, the need to comply with them did not perceivably drive or regulate current practices.

Autonomy was perceived to be greater in the private hospital setting than in the public hospital setting. Nursing staff noted that private surgeons had the capacity to *“dictate their own practice”* (F11, private theatre nurse), which was not necessarily driven by guidelines or hospital policy, as they were *“doing their own thing and renting the space” (*F11, private theatre nurse).

Surgical registrars also felt they had less prescribing autonomy compared to their medical registrar colleagues, as noted by an orthopaedic registrar when they compared their level of autonomy to that of their partner, a general medicine trainee:

*“Just the level of autonomy that physicians are granted from*, *like*, *two to three years out of [university]*, *in terms of writing a patient up… If you refer to a surgical unit*, *you get the opinion of a consultant*. *Everything that we do goes to the consultants*.*”* (F15, public orthopaedic surgeon)

#### 2.1: Guideline knowledge and awareness of limitations

Guidelines were considered to be useful, particularly to junior health professionals (Table 3), and their accessibility was considered an enabler for appropriate SAP prescribing. However, guidelines were also perceived as broad and insufficiently detailed to account for the expansive range of surgical procedures and any confounding environmental and patient-specific factors, and thus are a barrier to optimal SAP decision-making in complex scenarios (Table 3).

**Table 3:** Representative quotations from Theme 2 reflecting ‘Psychological Capability’ of the COM-B model.

Theme 2: Prescriber autonomy overrules guideline compliance.			
Subtheme	TDF Domains	Illustrative quotes	Barrier or enabler?
Guideline knowledge and awareness of limitations	• Knowledge • Memory, attention and decision processes • Behavioural regulation	*“No*, *because every patient is different*. *So*, *guidelines are guidelines*. *They’re not rules*. *So*, *they can be helpful*, *and they may*, *you know*, *stop you from giving them for five days*. *But*, *it’s not going to stop you from worrying*. *Especially*, *contamination*. *It’s not going to stop me*.*”* (F4, public colorectal surgeon)	Barrier
		*“I think this generation are very quick to refer to guidelines and look things up*. *I think they’re very good at accessing them*. *The sort of stuff that we used to carry around in our heads*, *it’s just not necessary anymore because you just have a look at the antibiotic guidelines and it’s there for you*.*”* (F4, public colorectal surgeon)	Enabler
		*“Even though there might be recognised guidelines*, *many surgeons have their own preferences; which they will override*, *and they might say*, *‘Yeah*, *I know there’s no evidence for antibiotics*, *but I want them anyway*.*’ Or*, *‘I know the guideline says use cefazolin or whatever*, *but I like ceftriaxone*.*’”* (F3, private anaesthetist)	Barrier
		*“I think the assumption for most of the surgeons would be that their practice that is deviating from these guidelines*, *does not deviate because they’re unaware of the guidelines*. *And us (pharmacists)*, *just letting them know that they exist isn’t going to solve the problem… The education of the surgeon is not necessarily the gap*.*”* (F3, private pharmacist)	
Competition as a means to regulate behaviour	• Behavioural regulation	*“So*, *where you feed that (audit data)… I mean*, *people in medicine*, *particularly doctors*, *are very competitive*. *So*, *you know*, *people don’t like to be down at the bottom of the pack… And*, *they want to do what’s best overall*. *So*, *if you show that they’re not meeting the guidelines*, *then they will work to get the results*.*”* (F3, private anaesthetist)	Enabler
		*“Neurosurgeons are very competitive. If you can show a unit that their infection rate is crap, they’ll try and do something about it”.* (F6, public neurosurgeon)	
		*“If you can benchmark and compare them (surgeons) to other people, that’s another way (to change practice).”* (F12, public pharmacist)	

There was awareness of national and local guidelines, but participants commonly highlighted the perceived existence of many gaps in the current evidence and how these warranted deviation from guideline recommendations. However, participants also noted that the rationale for deviation was not always documented. Common issues identified by participants could be mapped to specific targets for future educational stewardship strategies. These include timing of SAP administration (in relation to incision time), intra-operative re-doing, post-operative durations, the use of topical SAP, and SAP in relation to concurrent antimicrobial therapy and complex patients (Table 4).

**Table 4:** Commonly referenced gaps in current guidelines and evidence as per participants.

Examples	Quote (Focus group reference)
Timing	*“How late can you give it*? *It’s probably more relevant to the anaesthetist… Does it matter if it’s two minutes before the operation or twenty minutes before*?*”*. (F2, public anaesthetist)
Re-dosing	*“There’s a lot of debate about whether it should be three or four hours*.*”* (F10, public anaesthetist)
Weight-based dosing	*“I had an orthopod who was very concerned that… this was like a very serious operation*. *And*, *so gave the 3 grams for this not morbidly obese patient because they really wanted to give a good serious dose*.*”* (F10, public anaesthetist)
Post-operative durations	*“The guidelines say only one dose*, *but all surgeons that I work with want three doses for joints*.*”* (F3, private anaesthetist)
	*“We just continue antibiotics*, *you know*, *till the drains are out*. *We just do our own thing*. *It’s a case-by-case thing*. *It’s not particularly evidence-based… I wasn’t even really aware of those guidelines*.*”* (F5, public plastic surgeon)
Oral antibiotics	*“If you’ve had them on IV cefazolin*, *and you’re then going to prescribe them an [oral course of antibiotics]… If the wound’s dry and it’s not infected*, *why’re you giving someone antibiotics*? *I try and avoid that*. *But*, *it’ll usually be the boss to say*, *‘five days of orals’*.*”*(F15, public orthopaedic surgeon)
Complex patients	*“Being hit on the head in a car is different to being kicked in the face by a horse*, *for instance*. *So*, *they both might have similar fracture patterns but would have vastly different organisms and contamination*. *So*, *we’re naturally suspicious of protocol-driven care because I don’t think it’s nuanced enough currently to actually give us a meaningful answer*.*”* (F5, public plastic surgeon)
Topical antibiotics	*“There was a neurosurgeon I worked with in Adelaide who used to open the flucloxacillin*, *and put the powder into a pack*, *and sprinkle it on the wound*. *I was like*, *gosh*, *‘Mr*. *[surgeon]*, *is there any evidence for that*?*’ He goes*, *‘No*, *absolutely none*, *but I like to do it anyway*. *Makes me feel better’*.*”* (F3, private anaesthetist)
Concurrent antimicrobial therapy	*“If they’re on the ward*, *like ICU*, *and they’re on some mega*, *you know*, *fancy pants drug you’ve never heard of… what do we do then*? *Do we just give them some cefazolin*? *You know*, *is that the wrong thing to do or*, *you know*, *because…*. *We certainly don’t have mega penicillin that they give… and even though they’re not due for a next dose of meropenem*, *say*, *for another four hours*, *what do we do*? *I don’t know*.*”* (F2, public anaesthetist)

Guideline implementation was not standardised across the three hospitals. Education around guidelines, or lack thereof, was not a concern, as there was an assumption that most surgeons were aware of their guideline non-compliance (Table 3). Insufficient motivation to address this, or to intervene to promote guideline compliance, served as a barrier to optimisation of SAP prescribing. Inconsistent guideline implementation was seen as an accepted part of current practice.

#### 2.2: Competition as a means to regulate behaviour

It was noted by participants that surgeons are highly driven and competitive. Capitalising on these behavioural traits may be an enabler of improved SAP prescribing. Peer-to-peer and unit-to-unit comparisons of SAP prescribing may induce professional competitiveness and, as such, facilitate quality improvement initiatives (Table 3).

### Theme 3. Social codes of prescribing reinforce established practices

#### 3.1: Intra-specialty hierarchy rules

Surgical registrars, residents and interns were not comfortable modifying and ceasing SAP prescriptions without confirming the acceptability of such decisions with their consultants, indicating the influence of intra-speciality hierarchy on decision-making. Continuation of SAP orders in the absence of documentation, infection or evidence for use was perceived as a default practice, reinforced by preferences that have been passed down the hierarchy.

Prescribing behaviour was defined and regulated at a senior level, dependent on the surgical consultant’s preference as opposed to guideline recommendations. Participants noted, to generate changes in SAP prescribing practices, acceptance of interventions from the most senior medical level was required. Targeting SAP stewardship interventions at junior surgeons was not perceived as helpful, as their prescribing was heavily regulated by their seniors’ preferences, as opposed to externally-received advice advocating guideline compliance (Table 5).

**Table 5:** Representative quotations from Theme 3, reflecting ‘Social Opportunity’ of the COM-B model.

Theme 3: Social codes of prescribing reinforce established practices			
Subtheme	TDF Domains	Quotes (source)	Barrier or enabler?
Intra-specialty hierarchy rules	Social Influences	*“It’s (up to) the consultants*, *you can’t (change practice to single dose SAP)… They’ve got to be on board with it*. *Because*, *at the end of the day*, *you stop the antibiotics based on evidence*, *and something goes wrong–it will be your fault*. *It’ll reflect badly on you because the consultant will say*, *‘That’s not what I do*.*’ And*, *the guidelines are not enough to stand against a consultant who’s got very set ways*, *in terms of their antibiotic prescribing… Often*, *I’ll just go with what my seniors have told me because*, *you know*, *that’s what we do*.*”* (F14, public surgical resident/ registrar)	Barrier
Cross-specialty prescriber etiquette		*“We would resist*, *or not embrace*, *a nurse practitioner type approach to antibiotic use in theatre*. *We would welcome some input*, *but we wouldn’t necessarily agree or follow those instructions*. *It is… I know that it sounds somewhat snobby*, *but*, *if that was advice given to me by an ID physician*, *I would follow it because*, *I think*, *they come from a more informed position… If someone had just done a one-year course on a weekend to become a nurse practitioner to give advice on antibiotics*, *it just doesn’t sit well with me*. *I would back myself*.*”* (F5, public plastic surgeon)	Barrier
		*“(Prescriber) etiquette also extends to a surgeon (who) gets a formal ID consult on one of their patients… They’ll (ID) just recommend that the surgeon stops antibiotics… Some of the ID guys won’t cross off the antibiotics*. *They’ll just say*, *‘If they surgeon’s happy*, *I think*, *you could stop the IV [antibiotics]*. *I’m still going to leave it in your hands*.*’ I mean*, *it’s all very polite*.*”* (F13, private pharmacist)	Barrier

Orthopaedic registrars and residents noted that pointing out deviations from the guidelines when “*the boss wants to do something else*” was a form of “*challenging*” their boss (F15, public orthopaedic surgeon). Despite collegial support, they did not feel they were truly empowered to challenge the status quo and were expected to continue current SAP prescribing practices regardless of the deviation from evidence-based recommendations:

*“I think*, *the reality of the relationship with surgeons is that they don’t want to be challenged by their junior staff*. *People often encourage us to do* things, *like the College of Surgeons has got this escalation of stuff [framework]*. *It’s all BS [bullshit]*.*”* (F15, public orthopaedic surgeon)

Participants noted that, with time, the social codes are changing, with the new generation of younger surgical consultants demonstrating greater awareness of evidence-based recommendations. However, these surgeons still have to overcome established default practices such as the perceived standard practice of prescribing antibiotics for 24 hours post-operation.

“*I think*, *some of the younger consultants are up-to-date with [the evidence]… And*, *I think that’s what is going to change it–it’s just the next generation*. *But*, *the problem is*, *things get handed down*, *and*, *you know*, *because your boss did 24 hours [of post-operative SAP]*, *I have to do 24 hours*.” (F14, public surgical resident/registrar)

#### 3.2: Cross-specialty prescriber etiquette shapes decision-making

Surgeons, regarded as being at the top of the hierarchy, expressed reluctance to receive advice and feedback from those not at a similar level of seniority (i.e., a trained nurse practitioner), or stewardship recommendations (Table 5).

The way feedback is given to surgeons was perceived as constituting prescriber etiquette. AMS and ID specialties were not considered to be part of the surgical team, and, as such, their recommendations were to be considered but not mandatorily enforced. Participants noted AMS and ID physicians were less likely to actively modify or cease prescriptions out of professional etiquette and only provide a recommendation. The final decision remained with the surgeon (Table 5).

### Theme 4. Need for improved communication, documentation and data for action

#### 4.1: Poor documentation and communication

Communication at both inter- and intra-specialty levels was perceived to be lacking and a barrier to appropriate SAP prescribing and decision-making. In the peri-operative setting, the theatre environment was considered to be time-poor, hindering access to patient information and guidelines for antimicrobial clinical decisions. Participants noted an increasing availability of digital guidelines, which facilitates greater use among trainees, and may result in more guideline-compliant prescribing practices.

“*Most theatres have computers*. *Otherwise*, *we’ve got them (electronic guidelines) in our pockets at all times*.” (F14, public surgical resident/registrar)

Additionally, it was noted that documentation of timing for surgical incision and SAP administration was completed poorly, thus making it difficult for anaesthetists and surgeons to receive accurate feedback (Table 6).

**Table 6:** Representative quotations from Theme 4, reflecting ‘Physical Opportunity’ of the COM-B model.

Theme 4. Need for improved communication, documentation and data for action			
Subtheme	TDF Domains	Quotes (source)	Barrier or enabler?
Poor documentation and communication	Environmental context and resources	*“Probably the biggest caveat to any anaesthetic record is that it’s a record rather than a prescription*. *So*, *it’s often done in retrospect*. *So*, *they’ll give it and then they record it*. *So*, *if they’ve forgotten actually what they’ve given at what time… Sometimes*, *it might not happen*.*”* (F12, public pharmacist)	Barrier
‘Time-Out’ supports pre-operative communication; post-operative management is less standardised		*“They (surgeons*, *anaesthetists and scrub nurses) definitely use the time-out*. *Everyone uses the time-out*. *But*, *I’m not sure about the guidelines*.*”* (F11, private theatre nurse)	Enabler
		*“Orthopaedics will put in the operation notes–‘IV cefazolin 24hours*.*’ Which usually means intra-op and three doses*. *And that’s very clear*. *And that’s easy to denote on the med charts*. *And*, *other units*, *not necessarily–not as clear*.*”* (F14, public surgical resident/ registrar)	Barrier and enabler
		*“The number of times patients come back post-operatively for a check*, *and all they’ve had is a minor little dehiscence and the GP has just given them two-weeks of antibiotics… It’s completely inappropriate*.*”* (F7, private vascular surgeon)	Barrier
Data for action		*“Audit it [antibiotic use] and compare it to previous use where there has been prescription of antibiotics and there was no change*. *That would be the outcome required to convince surgeons to change their practice*.*”* (F15, public orthopaedic surgeon)	Enabler

#### 4.2: ‘Time-out’ supports pre-operative communication; post-operative management is less standardised

The ‘time-out’ process is part of the ‘universal protocol’[22] and ‘World Health Organization Surgical Safety Checklist’[23]. The ‘time-out‘ process has been adopted globally to provide reassurance of “*accurate patient identity*, *surgical site and the planned procedure*”[24, 25]. ‘Time-out’ was perceived to be an enabler for optimal procedural SAP prescribing and a successful workflow process that has been embedded into current practice and supports communication between the team. It also includes questions regarding “*allergies and what antibiotics patients are getting*” (F5, public plastic surgeon).

Participants noted the absence of a similar process in the post-operative setting and observed that post-operative plans “*won’t say anything about antibiotics*” (F11, private theatre nurse). If antibiotics were wanted by the surgeon, the plan was communicated poorly and usually handed over passively and verbally by the anaesthetist; for example, “*This surgeon wants three more doses*” (F11, private theatre nurse). Similarly, the discharge workflow and communications regarding the management of post-operative complications and/or infections between GPs, pharmacists and surgeons were perceived as being sub-optimal.

Participants agreed that inclusion of a discussion about SAP duration as part of the pre-operative time-out, or the addition of a post-operative ‘time-out’ or checklist, would serve as a potentially useful intervention that would enhance the clarity of the documentation and planning of SAP duration.

“*I would think the best place to do it is in the time-out in the theatre*, *and then trying to change the paperwork to that… So*, *then you can say*, *‘Right*, *this can be written for 24 hours or this can be a one-off*.*’ You get clear on that*.” (F11, public theatre nurse)

#### 4.3: Data for action

Many participants stated that local data on appropriateness of antimicrobial prescribing and outcome data such as surgical site infections, was of greater interest to surgeons, and an enabler of SAP optimisation. It was perceived that the use of local data was more likely to generate behaviour change and improve SAP prescribing (Table 6). Many participants expressed the view that the guidelines and the underlying evidence base weren’t tailored to their unique patients and setting; they wanted local data to inform their practice. However, it was also noted that there was limited funding for localised data collection and dissemination of feedback.

### Theme 5. Fears and perceptions of risk hinder appropriate SAP prescribing

#### 5.1: Fear of infections

Participants expressed many concerns and fears in relation to SAP prescribing, and how this impacted upon both the patient and themselves. Overall, all fears illustrated in Table 7 were barriers to appropriate SAP prescribing. There was awareness of how inappropriate prescribing may contribute to patient harm and the development of multi-drug resistant organisms (MDROs), but the risk of development of MDROs and adverse consequences from antibiotic overuse was perceived as being outweighed by the risk of perceived underuse causing direct patient harm, particularly in the form of not preventing surgical site infections (Table 7).

**Table 7:** Representative quotations from Theme 5 reflecting ‘Automatic Motivation’ of the COM-B model.

Theme 5: Fears and perceptions of risk hinder appropriate SAP prescribing.			
Subtheme	TDF Domains	Quotes (source)	Barrier or enabler?
Fear of infections	Emotion	*“I think the problem is that an infection is a nightmare*. *An infected joint is a catastrophe… It could end up with the whole joint being removed*. *The patient having amputation… I can completely understand why a surgeon would want more than one dose*.*”* (F3, private anaesthetist)	Barrier
		*“They don’t care about the multidrug resistant bugs… They care about what looks bad on their [statistics]… What looks bad on their [statistics] is when they get a post-op wound infection*. *But*, *these patients with multidrug resistant bugs*. *The problem is down the track*, *it’s not (an issue)*.*”* (F15, public orthopaedic surgeon)	Barrier
Varied risk perceptions across specialties		*“I think*, *as colorectal surgeons*, *most of us… if we had a localised surgical site infection*, *antibiotics are not the first thing we would use*. *We would open up the wound or drain any pus that’s around*. *And*, *most of the time… if I had a post-operative wound infection which was superficial on the abdomen*, *I probably won’t use antibiotics*. *So*, *I think*, *we don’t usually use antibiotics*. *Whereas*, *plastics on the other hand… you know*, *if you went and rubbed a bit of faeces on to one of their skin grafts (laughing)*, *it’ll be horrifying*. *We do flaps in the anus*, *and we don’t use long*, *extended antibiotics for that*.*”* (F4, public colorectal surgeon)	Barrier and enabler
		*“I think*, *21 days of [piperacillin/tazobactam] is the longest*. *We’ve had some head and neck patients on maybe like three or four weeks of IV cefazolin because everyone chickened out of taking it off*. *We’ve had patients started back on IV cefazolin because they looked a little bit red with no objective evidence of any infection*.*”*(F14, public surgical resident/registrar)	Barrier
Fear of litigation		*I think*, *a lot of the surgeons are really*, *genuinely scared about being sued*. *I’m sure that drives practice… I think it makes surgeons very reluctant to undertreat infections because they’re the ones who’re going to cop it if the patient has a complication*.*”* (F8, public urological surgeon)	Barrier
		*“I think (that’s) what’s a major contaminant in the space… is the behaviour of Medibank Private*, *who are looking to not fund readmissions and problems for post-operative infections*, *etc*. *I think that is a significant pressure that some surgeons will feel*, *and will mitigate against*. *So*, *someone will say*, *‘Yeah*, *I want to give more antibiotics*. *I’m worried about (infections)*.*”*(F4, public colorectal surgeon)	Barrier
Risking career progression and job security		*”We’re working in an environment now where making criticisms of your colleague’s practices is extremely troublesome*. *And*, *I think*, *my experience*, *as someone who gets frustrated and tends to make comments*, *is being told to shut up*.*”* (F15, public orthopaedic surgeon)	Barrier
		*“I think*, *if you made a pattern of behaviour*, *where even if you’re advocating for a patient but you’re asking some questions that were seen to be undermining the authority of the surgeon*, *depending on personality*, *or just creating a headache for them*, *in that*, *‘Who’s this registrar*? *Always asking me these tough questions that I don’t know the answer to*.*’ I think you could get into a position where*, *you know*, *it affects your relationship with that surgeon… If you’re annoying and ask stupid questions*, *that slowly will affect their view*. *They’re not going to respect you necessarily*, *for asking the tough questions*. *They just will consider you a hassle*.*”* (F15, public orthopaedic surgeon)	Barrier

Surgeons reported attributing blame for MRDOs to other health professionals, such as GPs, with little reported acknowledgement of their own contributions and prescribing behaviour. Other health professionals were perceived as having greater responsibility for the problem.

“*I’ve heard (other) surgeons say*, *‘Oh*, *it’s not our fault*. *It’s the bloody GPs*, *you know*, *prescribing [antibiotics] in the community*.*’ No one wants to take blame for that problem*.” (F15, public orthopaedic surgeon)

To facilitate prescribing behaviour change, it was suggested that the discussion around SAP appropriateness needs to shift from highlighting the lack of evidence for prolonged duration of SAP to focusing on the potential harms of inappropriate antibiotic use, emphasising the importance of appropriate use and responsibility for enhancing patient care.

“*Our message up till now has been there’s no great evidence of benefit of continuing the [antibiotics]*. *We haven’t really sold the message of what’s the risk of harm*. *Because*, *if there’s none*, *then it’s*, *‘3 days of cefazolin*, *who cares*?’” (F13, private pharmacist)

#### 5.2: Varied risk perceptions across specialties

Risk perception varied across the surgical specialties and was both a barrier and an enabler to appropriate antibiotic use. Colorectal surgeons were less likely to use antibiotics for a post-operative infection, whereas plastics surgeons were considered to be overcautious, prescribing prolonged durations (Table 7).

Surgeons were less likely to be aware of antibiotic-related adverse events such as *Clostridioides difficile* infections, and less likely to factor the risk of adverse events into their prescribing decisions.

*“Clostridium [Clostridioides] difficile is not an orthopaedic problem area*. *Clostridium [Clostridioides] difficile does not stand in an orthopaedic bed cart [not an orthopaedic admission] and it’s not a headache for us”*. (F15, public orthopaedic surgeon)

#### 5.3: Fears of litigation

The fear of litigation from patients for post-operative complications drives antibiotic overuse as a preventative measure, and this perception emerged across all participating cohorts. Negative experiences such as previous complications and litigations also appeared to encourage the over-prescribing of SAP (Table 7).

Surgeons perceived that there was a ‘backlash’ for post-operative complications and SSIs from many sources: the hospital, surgical colleagues, other medical professionals and health insurance companies. Avoidance of this outcome appeared to be a justification for overuse of SAP in the post-operative setting.

Health/medical insurance companies influence how risk is perceived and managed in the private setting. Clinicians perceive insurers as being resistant to funding readmissions prompted by post-operative SSI occurrence. This may drive an overcautious approach to SAP prescribing (Table 7).

#### 5.4 Risking career progression and job security

Challenging senior consultants about antibiotic prescriptions was not considered to be worth the perceived associated risk of jeopardising their career and relationship with that surgeon, and therefore serves as a barrier to optimal SAP prescribing (Table 7). Junior surgeons were less willing to dispute their seniors’ sub-optimal SAP decision-making, out of fear of appearing *“difficult”*, “*annoying”* and *“troublesome”*‘ (F15, public orthopaedic surgeon), which they believed would consequently affect their career prospects.

### Theme 6. Lack of clarity regarding roles and accountability

#### 6.1. The buck stops with the surgeon; ownership required

Ultimately, the responsibility for SAP lies with the surgeon as “*the buck stops with them*” (F13, private pharmacist). Post-operative complications were viewed as a serious issue and addressing these was considered, by default, to be the surgeon’s responsibility.

Participants reported that responsibility for SAP prescribing in general was transferred to the most senior professional (surgeon or anaesthetist) in the theatre at a given point in time. However, the multiple roles required for clear decision-making around SAP prescribing pre-, intra- and post-operatively are less clearly defined, with adverse implications for accountability for these decisions.

When other professionals were involved in SAP prescribing, the responsibility associated with each role varies, creating further confusion amongst the team; and was considered as a barrier to appropriate SAP prescribing. Ideally, clarification and ownership of these roles and responsibilities is required for all professionals in the surgical setting. This may enhance both prescriber accountability and correct and timely referral to the appropriate decision-maker for queries pertaining to SAP. Participants noted that such clarity would support optimisation of antimicrobial use in the surgical setting (Table 8).

**Table 8:** Representative quotations from Theme 6 reflecting ‘Reflective Motivation’ of the COM-B model.

Theme 6. Lack of clarity regarding the roles and accountability			
Subtheme	TDF Domains	Quotes (source)	Barrier or enabler?
The buck stops with the surgeon, ownership required	• Social/ professional role and identity • Beliefs about Capabilities • Beliefs about Consequences	*“Well*, *they (surgeons) are (responsible)*. *And*, *part of the reason for that is because the buck stops with them when it all turns horrid… It’s ultimately theirs… There’s a reason why they get to make the call*.*”* (F13, private pharmacist)	Barrier
		*“We’ve got a good thing in place at the moment where the anaesthetists are owning it intraoperatively*, *and*, *I think*, *postoperatively*, *it’s put back on to the teams*, *which is then where we see the major variance in practice*.*”* (F12, public pharmacist)	Enabler
		*“I think someone needs to own it*. *So*, *I would say most hospitals… whether it’s the anaesthetist or surgeon*, *someone has to own it*. *If you give it to too many people*, *no one’s going to own it*, *and no one will do it*. *So*, *they need to have a champion*. *So*, *whether it’s anaesthetists in theatre… for us*, *here*, *it’s quite anaesthetic-driven*. *Which has been good*.*”* (F12, public pharmacist)	Enabler
Passive prescribing hinders accountability and SAP cessation		*“Most of the time*, *they’re (surgical consultants) not really aware of what their residents and registrars are charting*.*”* (F10, public anaesthetist)	Barrier
		*“But*, *most of the teams here*, *because they work together for so long*, *it is*, *you know*, *practice of what they’ve always done*. *So*, *I guess*, *it’s a conversation that they’ve (anaesthetists and surgeons) had previously*, *and it is assumed that that’s what they just keep going with*.*”* (F11, private theatre nurse)	Barrier and enabler
		*“The default for the resident is to continue with the status quo until someone tells them it’s okay to stop*. *So*, *again*, *the pharmacist will often only get access to the resident*, *who won’t know what to do necessarily*, *and they’ll continue it as a default*. *It’s access to the senior decision-makers that is often the gap*.*”* (F10, public anaesthetist)	Barrier
Capacity for role expansion of pharmacists and nurses.		*“There is orthopaedic prophylaxis that is charted by the PAC pharmacists in pre-admission clinic*, *which is again very protocol driven…And*, *that includes any post-operative prophylaxis*. *I think*, *it’s a very collaborative model*. (F12, public pharmacist)	Enabler
		*We do rounds with the pharmacists…They question us about everything…They (pharmacists) are very helpful*. (F9, public cardiothoracic surgeon)	Enabler
		*“A lot of the antibiotic decisions go over the pharmacists’ heads because they don’t have that real clinical insights as to the decisions that were made*. *I quite often have discussions with the pharmacists about why we’ve chosen antibiotics for plastics*, *for head and neck patients*, *and they just don’t have any understanding of*, *like the intraoral communication and what difference that makes…”* (F14, public surgical resident/ registrar)	Barrier
		*“I think*, *nurses are much better at following guidelines…And*, *ward pharmacists*. *If there was a very clear statement around these (antibiotics)_ in the surgeries*, *that they should or shouldn’t (be prescribed)*, *then they will flag that up”* (F10, public anaesthetist)	Enabler

#### 6.2: Passive prescribing hinders accountability and SAP cessation

Prescribing for SAP appeared to be undertaken passively by anaesthetists in the peri-operative setting, and by surgical residents/registrars and interns in the post-operative setting.

Decisions regarding SAP depend upon the surgeon; however, anaesthetists, who have an established role in SAP administration, are expected to have SAP prepared prior to surgery, where decisions may have not been finalised. This places them in an unclear position of responsibility; and serves as a barrier to appropriate SAP prescribing.

Discussion regarding the initiation of procedural SAP appeared to be part of the current workflow and ‘time-out’ process, with anaesthetists noting that they would commonly prescribe and administer SAP on behalf of the surgical consultant. This routine would be based upon previous discussions regarding the surgeon’s preference, as opposed to guidelines. Some surgeons deferred SAP decision-making to the anaesthetist, which was considered successful in improving guideline compliance (Table 8).

While surgeons were identified as being responsible for SAP, they were also perceived to be abrogating responsibility to junior registrars without clearly communicating their expectations (Table 8).

There did not appear to be a regular process or pathway for prompting discussion about cessation and/or continuation of post-procedural SAP. Consequently, junior surgeons passively prescribed SAP on behalf of their consultants without further discussion of a plan.

Cessation of SAP requires active intervention, and, by default, SAP was commonly and passively continued in the post-operative setting until someone confirmed cessation with the consultant. Junior surgeons were reluctant to question their senior’s decisions and consequently reluctant to change or cease post-operative SAP.

“*As a resident… they (nurses and pharmacists) do ask you*, *‘Should we be stopping this*?*’*, *or*, *‘How long are we giving this for*?*’ Like*, *it’s not within my power to say*, *‘Just stop it*.*’ I have to ask someone else*.*”* (F15, public orthopaedic surgeon)

This perceived lack of leadership for SAP cessation was noted by participants across all professions.

#### 6.3: Capacity for role expansion of pharmacists and nurses

Participants reported that pharmacists were not regularly present in the operating theatres across all sites included in our study, and therefore were perceived to have minimal influence of SAP decision making. One site described the novel expansion of the pre-admission clinic pharmacists’ role in response to poor adherence of their guidelines. The procedural SAP was charted and administered via the anaesthetic record and pharmacists undertook an active role in the charting of post-operative doses (two additional doses of cefazolin 2 grams every eight hours, for a total of 24 hours) for orthopaedic hip and knee arthroplasty as per their local protocol (Table 8). Ultimately, changes could be made by the orthopaedic unit. The capacity building undertaken for this pharmacy role expansion was perceived to be beneficial, as it ensured correct weight-based dosing and SAP duration. This intervention supported local protocol compliance which had been endorsed by the hospital and orthopaedic unit, thus serving as an enabler to SAP optimisation and standardisation.

Pharmacists were noted for their active role in reviewing post-operative SAP when patients returned from theatre. This was generally perceived as helpful by other professions (Table 8). However, as previously discussed, the final decision was dependent upon the junior doctors following up on the pharmacists concerns and querying these with their surgical consultant.

The pharmacists’ scope of practice and knowledge of antibiotics decision-making was queried by surgical residents, in which one stated that pharmacists lacked *“real clinical insight”* (F14, public surgical resident/ registrar) into their decision-making and were frustrated by their constant need to justify their decision-making to pharmacists. This served as a barrier to expanding the pharmacist roles and capacity for SAP optimisation.

The role of the pharmacist varied across the included hospital sites. One of the public hospitals noted the pharmacy departments’ strong clinical presence on the wards had led to an embedded practice where all prescriptions must be reviewed by a pharmacist prior to discharge (F9, public pharmacist). Another site, a private hospital, highlighted the difficulties of timely access to surgeons and subsequent reduced ability to providing accurate post-prescription review and feedback (F13, private pharmacist).

Theatre nurses were noted to have multiple roles in the operating room. Their role regarding SAP was perceived to be highly protocolised. Experienced nurses were perceived to be more confident in their ability to prompt anaesthetists and surgeons regarding pre-operative SAP administration. Nurses did not feel empowered to have an active role in improving SAP as they were not in charge of administration and documentation;

*“I don’t think we’re in charge of documenting when it can be given*, *to be honest with you… That power is taken out of our hands as to when it can actually be administered”* (F11, private theatre nurse)

SAP decision-making in the post-procedural setting was unanimously perceived as beyond a theatre nurses’ scope of practice (F11, private theatre nurse). However, they did acknowledge the importance of their role as key communicators between theatres, recovery and ward nurses to ensure that the surgeons plan for antibiotics was followed up on.

Whilst decision-making was beyond their scope of practice, nurses believed they could become more actively engaged in the process if an intervention was of similar format to the ‘time-out’ process, in which they felt comfortable in following the prompts of a check-list and/or protocol, as it had been endorsed as hospital policy and their questioning of the surgical team appeared justified (Table 6, Subtheme 4.2).

## Discussion

Results from this study have highlighted the complexity of SAP prescribing behaviour and the need to understand these influences to inform the development of targeted AMS interventions. Our study has identified six themes that reflect key issues relating to the complex nature of decision-making around SAP prescribing. These themes were developed using the TDF[16] and COM-B[18] models, with the majority of themes identifying barriers to appropriate SAP prescribing.

### SAP is a low priority for surgeons

The notion that SAP is a low priority for surgeons was also highlighted by Broom *et al*[26] in their qualitative study involving Australian surgeons and anaesthetists. In this study, the authors’ focus was largely on the lack of priority for re-dosing intra-operative SAP, which was also illustrated in our analysis. Antibiotic management was perceived as a peripheral role by surgeons in a UK ethnographic study[27], whilst a lack of motivation and time to develop non-technical skills was also highlighted in a Sri Lankan qualitative study involving fifteen surgeons[28].

This lower prioritisation notably reflects the current poor uptake of SAP guidelines. The Therapeutic Guidelines[29] are the nationally endorsed and recommended guidelines in the AMS Clinical Care Standards[13]. They have also recently been updated since the completion of this research[29]. Guidelines were commonly identified as a reference point for SAP decision-making, with junior surgeons perceived to be more likely to access them. However, their limitations were widely acknowledged (Table 3), and prescriber preferences and autonomy were commonly seen as justifying non-compliance.

Broom *et al* [26] proposed that ‘improvisation’, and social and professional ‘norms’ work against guideline compliance. Similarly, we found there was a perceived need among prescribers to exercise their autonomy, and to improvise and deviate from recommendations. We believe that engagement with surgeons to address their perceptions about guidelines, and to gain consensus regarding recommendations, at both a national (specialty boards) and local (local guideline committees) level, is critical for the improvement of SAP prescribing. These findings also highlight that lack of knowledge about SAP is not necessarily a key barrier that drives inappropriate SAP prescribing. Education regarding the evidence that underpins guidelines is important but does not appear to be solely effective. An effective AMS intervention should be multi-faceted and consider the multiple additional factors that influence prescribing behaviour. Table 9 provides a summary of recommendations, mapped to the TDF and COM-B frameworks, for the optimisation of SAP decision-making and prescribing based upon our findings.

**Table 9:** Recommendations for change to optimise appropriate SAP decision-making and prescribing.

COM-B	Theme	Subtheme	Recommendations for change
Physical Capability	Low priority for surgical antimicrobial prophylaxis prescribing skills.	Surgical technique of greater importance	• Provide targeted education and training on principles for SAP and AMS as part of orientation training. • Increase surgeon engagement in SAP decision-making and prescribing.
		Deskilling surgeons	
Psychological Capability	Prescriber autonomy overrules guideline compliance.	Guideline knowledge and awareness of limitations	• Continue to increase accessibility of guidelines in the operative setting. • Increase engagement of surgeons and anaesthetists in guideline development. • Mandate documentation for rationale of guideline deviation.
		Competition as a means to regulate behaviour	• Conduct and feedback SAP prescribing audit data to departmental heads to facilitate benchmarking at multiple levels—consultant, surgical unit, hospital, state.
Social Opportunity	Social codes of prescribing reinforce established practices	Intra-specialty Hierarchy Rules	• Target AMS interventions at senior surgical consultants. • Engage with senior surgical consultants for the development and implementation of quality improvement initiatives such as AMS.
		Cross-specialty prescriber etiquette	
Physical Opportunity	Need for improved communication, documentation and data for action	Poor documentation and communication	• Promote standardisation of SAP workflow and documentation, specifically; - Indication for procedural and post- procedural SAP; - Duration of post-procedural SAP, and differentiation between SAP and antibiotics for treatment of actual infection; and - Details regarding antibiotic management in the hospital discharge summary for GPs and plans for re-referral. • Modify the ‘time-out’ process to include discussion of post-operative SAP. • Develop tailored clinical decision support systems and prompts for SAP.
		‘Time-Out’ supports pre-operative communication; post-operative management is less standardised	
		Data for action	• Engage with surgeons to identify relevant quality indicators. • Support ongoing SAP prescribing audits. • Increase capacity for auditing of outcomes in relation to quality of SAP prescribing.
Automatic Motivation	Fears and perceptions of risk hinder appropriate SAP prescribing.	Fear of infections	• Collect local outcome data that supports evidence-based SAP, rather than prolonging SAP out of fear of infections. • Highlight the potential harms to the patient from inappropriate antibiotic use to promote AMS initiatives.
		Varied risk perceptions across specialties	• Tailor AMS interventions that are reflective of the surgical specialties’ niche risk perceptions, e.g., recommend alternative non-antibiotic creams and ointments post-plastic surgical procedures.
		Fear of litigation	• Gather support from hospitals at an executive/policy level to enable surgeons to prescribe evidence-based SAP. • Undertake further research into the impacts and influence of private health insurers on antimicrobial prescribing.
		Risking career progression and job security	• Increase awareness of current fears and issues facing junior surgeons. • Collaborate with existing programs such as the RACS action plan to build a ‘culture of respect’.
Reflective Motivation –	Lack of clarity regarding the roles and accountability	The buck stops with the surgeon, ownership required	• Promote collaboration between surgeons and anaesthetists for the development of a SAP pathway or workflow that defines both professionals’ roles pre-, intra- and post-operatively to increase role clarity and accountability for SAP. • Promote electronic prescribing and AMS workflow to support greater prescriber accountability.
		Passive prescribing hinders accountability and SAP cessation	
		Capacity for role expansion of pharmacists and nurses.	• Development and training for emerging roles of nurse and pharmacist prescribers. • Development of Enhanced Recovery After Surgery (ERAS) pathways that include both pre and post-procedural SAP and AMS principles across the operative setting. • Partnered/collaborative SAP prescribing models

### Hierarchy influences prescribing behaviour

Our findings about hierarchy heavily influencing prescribing reinforce the existing literature [26, 28, 30–35]. Charani *et al*[35] proposed that medical hierarchy necessitates ‘prescriber etiquette’, whereby “unwritten but widely accepted cultural rules”[34] influence how health professionals prescribe and interact with other health professionals regarding SAP prescription, administration, monitoring and cessation. The preference for ‘non-interference’ is demonstrated in our study, where participants reported perceiving AMS/ID physicians as being reluctant to actively cease antimicrobials and alternatively provide recommendations that may not be implemented as this is dependent on the surgeons final decision.

Surgical hierarchy also influences how receptive surgeons are to feedback. Feedback was considered to have higher impact if delivered by health professionals of ‘similar status’, i.e., anaesthetists and ID consultants, as opposed to pharmacists and nurses. Thus, we recommend that future AMS interventions and strategies are tailored to suit the targeted audience and their level of hierarchy (Table 9).

### Need for greater standardisation, documentation and communication

Our study highlighted a need for greater clarity of roles and responsibilities for SAP prescribing and identified many gaps in communication and documentation across the patient’s surgical journey. The quality of the information on SAP prescribing that is recorded in the medical notes can vary between different specialities.

The standardisation of the SAP workflow and documentation practices supports accountability for all professionals in the surgical setting. Standardising communication regarding and documentation of the specifications of both procedural and post-procedural SAP prescribing would help rectify the routinely observed absence of this information in the medical notes, and help facilitate better auditing, education and feedback strategies.

The uptake of electronic medical records (EMR) and prescribing has the potential to support prescriber accountability as the prescriber’s name is more visible as opposed to an uninterpretable signature on the current paper medical charts and documentation. The Rawson *et al* [36] systematic review suggests adoption of clinical decision support systems (CDSS) may improve evidence-based antimicrobial use. We advocate for the development of CDSS and prompts that are tailored to SAP management for the optimisation of SAP prescribing (Table 9). The increasing uptake of EMR also facilitates the standardisation of documentation for SAP, in particular defining duration, thus potentially minimising inappropriate passive prescribing practices. The role of EMR was not a focus of our study as none of the hospitals included had adopted EMR and computerised physician order entry (CPOE) at the time of the focus groups.

Clinical pathways such as enhanced recovery after surgery (ERAS) protocols are well-established multi-disciplinary approaches that have been successfully implemented across hospital services worldwide [37, 38]. SAP administration is a common component of ERAS Society recommendations for peri-operative care[37, 39, 40] However, clarification of post-operative antibiotic management is omitted. Expansion of existing pathways to include this would facilitate further standardisation of post-operative antibiotics, which as per recommendations [29] would be infrequent and for a defined set of surgical procedures (e.g. laryngectomy, total knee arthroplasty and orthognathic surgery). A review of ERAS in relation to antibiotic use by Moffat-Bruce et al[38] proposed that the application of surgical guidelines within ERAS protocols and its expansion into the EMR environment would improve clinical workflow and reduce errors. Specific examples include: the development of order sets and dynamic orders for specific antimicrobial courses with procedure specific durations and stop times to be entered prior to surgery.

Modifying the time-out process, which was unanimously regarded by prescribers as a successful intervention, to better incorporate discussion of and planning around SAP prescribing would further enhance prescribing practices by addressing and rectifying some of the communication and information gaps in current practice that were highlighted by participants. Facilitating inclusion of SAP discussion within the surgical workflow through a check list or other prompt within the time-out process, and improved documentation, supports prescriber accountability, and increases role clarification for the rest of the team regarding the initiation, continuation and cessation of SAP (Table 9). Greater prescriber accountability will also reduce passive prescribing and delegation of SAP management to junior staff, which was also noted by the Charani *et al* [27] ethnographic study of a surgical ward round in a 1300-bed multisite healthcare organisation in the UK.

Engaging with surgeons and the operative team to identify important quality indicators will facilitate data collection that supports meaningful feedback and action for change and quality improvement (Table 9).

The efficacy of clinical pathway structures and systematic approaches to managing SAP dosing and duration could be supported by new and emerging roles of nurse and pharmacist practitioners. Evidence demonstrates that non-medical prescribing by nurses and pharmacists in the UK is as safe and effective as doctor prescribing[41]. A recent systematic review of fifteen studies demonstrated low to moderate evidence that pharmacists can prescribe to the same standards as doctors.[42] In Australia, non-medical prescribing has been adopted with various prescribing models across multiple specialties: dentists, nurse practitioners, midwives, podiatrists and optometrists[43]

Nurse practitioners are an established profession in Australia, with approximately 1,800 registered in 2019 [44]. Nurse practitioners are legally qualified to prescribe antimicrobials, which account for approximately one-third of their prescriptions [45]. Nurse practitioners are more experienced in advanced practice frameworks, demonstrate leadership skills[46], and are familiar with antimicrobial stewardship and the operative environment[47–49], thus we would consider them to have a positive impact in the development and implementation of an SAP optimisation intervention. Barriers that prevent the success of such initiatives exist and largely reflect the identified issues regarding hierarchical influences of SAP prescribing. Engagement and collaboration with surgical specialties is essential for the development and implementation of such initiatives.

Pharmacists currently do not have prescribing privileges in Australia. There is currently no research in Australia comparing the role of peri-operative pharmacists or nurses charting SAP in comparison to their surgical and anaesthetic colleagues. However, there are many examples in the current literature where clinical pharmacists have been involved in the charting of medications in Australian hospital settings[50–54]. One Australian hospital adopted a partnered-pharmacist charting model in their general medical and emergency chart stay units as an alternative to medical prescribing. The study identified that partnered charting between medical staff and pharmacists significantly reduced inpatient medication errors (standard medical charting: 78.7% vs partnered pharmacist charting: 3.7%, p<0.001)[50].

Another Australian hospital demonstrated a statistically significant reduction in medication errors when they compared their intervention of introducing a ‘PeRiopErative and Prescribing’ (PREP) pharmacist to join an elective surgery multidisciplinary team to improve accuracy of medication history, inpatient prescribing and discharge prescriptions[54]. The small study (n = 104) demonstrated significant benefits in medication history (9% [n = 53] vs. 96% [n = 51]; P < .001) and inpatient prescribing in the intervention arm, with fewer errors than the control group (0.64 vs. 1.31 errors per patient; (P = 0.047)[54].

Many barriers to pharmacist prescribing exist. A scoping review of these barriers by Zhou *et al* [55] revealed three key themes that should be factored in to the current debate: (1) the socio-political context; (2) resourcing issues; and (3) prescriber competence. We advocate for the ongoing exploration of the expansion of the peri-operative pharmacists and nurses’ role in prescribing and collaborative partnered charting models. An intervention of this nature would require extensive research into capacity for training and development of such roles and addressing the barriers Zhou *et al* [55] described; in particular implementation issues such as policy development, engagement with stakeholders, especially surgeons and anaesthetists.

### Fear of consequences to patient

The fear of causing direct harm to the patient by not preventing infections was a driving factor for the prolongation of SAP. Similar to our study, a US qualitative study of thirty inpatient physicians[32] highlighted that the potential adverse effects of antimicrobial use and concerns about AMR were not prominent considerations in SAP decision-making. Prevention of SSIs was considered a more important and tangible outcome.

Perceptions regarding risk notably varied between the health professions and at the surgical craft group level. Comparisons have been reported between medical and surgical antibiotic decision-making[56], but not across the surgical craft groups. Further analysis of our qualitative data is warranted and may reveal specialised targets for AMS initiatives that specifically respond to niche surgical craft groups concerns.

The perceived influence of private health insurers on a surgeon’s autonomy was considered a barrier to optimal SAP prescribing and promoted prolonged SAP as a measure to prevent infection and avert blame for causing infection. Medical overtreatment such as prolonged antibiotic use is considered common practice, and fear of being reported for malpractice was identified as the most common reason for this (84.7%) in a US survey of 2,106 physicians[57]. Further research into the impact of health insurance companies and their influence on SAP decision-making will be beneficial for informing AMS interventions at a national health policy level.

Of noted concern, many participating surgeons felt that they could not speak up against their colleagues or seniors regarding antibiotic management due to fear of a negative impact on their career progression. In 2015, the Royal Australasian College of Surgeons (RACS) published an Action Plan[58], as an initiative to build a ‘culture of respect’ in order to improve patient safety; however, some participants questioned the success of such initiatives. Collaboration with stakeholders across RACS, other surgical craft group specialities, junior surgical fellows and trainees with AMS and ID specialties is required for the development of effective AMS initiatives that will address ongoing issues of hierarchy and fear of career regression, thus supporting prescriber accountability for SAP decision-making.

### Strengths

The use of qualitative methodologies to develop a greater understanding of complex prescribing behaviours is well established in the current infectious diseases and AMS literature [26, 27, 32, 34, 56, 59, 60]. Over time, health care services have shifted to a multi-disciplinary collaborative model. Therefore, we included surgeons, anaesthetists, theatre nurses and pharmacists across the public and private hospital sectors to better understand the broader context of SAP decision-making. Our study is the first to explore observations and perceptions regarding SAP decision-making across a range of health professionals and surgical specialties. Previously, a qualitative study focusing on antibiotic decision-making in surgery was undertaken in the UK [27]. Following on from this, Australian qualitative research including twenty in-depth semi-structured interviews with surgeons and anaesthetists has been published[26, 59].

Our findings have been developed through the application of theoretical frameworks. The adoption of theoretical frameworks provides structure to the design and analysis of qualitative research and is well-described in the current literature [61–64]. McIsaac *et al* (37) proposed that the combination of the TDF and BCW delivered a stronger theoretical approach to implementation research. However, the literature suggests there are limited published interventions targeting antimicrobial use that use behavioural theory[65, 66]. Michie *et al*[67] suggested the consideration of human behaviour theories may improve an intervention’s efficacy.

The application of social and behavioural sciences assists in improving understanding of current behaviours and problems relating to AMS[68] and supports the adoption of a theory-driven approach to AMS intervention design. We propose our findings provide direction for theory-informed AMS interventions that account for the complexities of the surgical setting. Table 9 provides a summary of our recommendations to improve practice in relation to the barriers and enablers identified in our analysis. Further exploration of the current qualitative dataset using the BCW will support greater translation of findings on barriers and enablers of SAP decision-making in the form of targeted interventions and policy initiatives [17].

Proposed triangulation with existing quantitative data, such as the Australian Surgical NAPS[4], will validate our findings and further illuminate the current state of SAP prescribing in Australia. Such methodologies could be adapted by others to support their research on SAP prescribing.

### Limitations

Our study was conducted across only three hospitals within the Australian state of Victoria, and, therefore, the opinions represented may not be truly representative of all surgeons, anaesthetists, pharmacists and theatre nurses in Australia. The recruitment of surgeons for focus groups was time consuming. Focus groups with a minimum of four participants were organised and, in few cases, when a participant could no longer attend at the last minute, we proceeded with those present. Whilst we have been able to include numerous surgical specialties, not all have been represented in this research. Engagement with surgeons in AMS research has been identified as suboptimal. We found that engaging with surgical consultants in the private hospital setting to be challenging. The reasons for non-participation were not collected and deemed beyond the scope of this study. We believe these findings were not unique to our study, as other Australian[59] and UK[27] qualitative studies have also demonstrated poor engagement of surgeons in SAP prescribing, guideline compliance and AMS research.

A limitation of utilising focus group methodology is the inability to guarantee equal representation of all participants within the group. We acknowledge that some participants are more vociferous than others and therefore their own personal views may project across the focus group. In an attempt to minimise this, the sampling and recruitment of the focus groups was designed to ensure that participants felt comfortable to voice their opinions. If junior staff did not voice their thoughts in the focus group, follow up interviews were planned to be conducted if necessary. An additional focus group solely for surgical registrars and residents was also included to promote participant comfortability.

## Conclusions

Our study identified six key themes that complement and build upon the existing literature and can be applied to enhance understanding of the enablers and address barriers of SAP optimisation, and support development of theory-informed AMS interventions. Many challenges remain for the optimisation of SAP across multiple surgical settings. We advocate for the development of initiatives that aim to enhance the prioritisation of and accountability for SAP, via the standardisation of workflow, roles and documentation across the peri-, intra- and post-operative settings. Interventions should address the underlying social issues that influence SAP decision-making, such as professional hierarchy, varied risk perceptions and fears about patient harm, litigation and career regression. Increased engagement of surgeons and the operative team in the development of data collection practices, local policies and guidelines will likely enhance the uptake of quality improvement actions and minimise the gap between evidence-based and current practices. Future research across different national and international hospital settings and additional surgical specialties will further build upon this exploration of SAP decision-making and strengthen the need for theory-driven AMS interventions.

## Supporting information

S1 Appendix Focus group question and discussion guides. - Tables A and B.
(PDF)

S2 Appendix Themes and subthemes mapped to the COM-B Model and Theoretical Domains Framework. - Table C.
(PDF)
