# Supplementary material for: Influences on surgical antimicrobial prophylaxis decision making by surgical craft groups, anaesthetists, pharmacists and nurses in public and private hospitals
Source: PLoS One. 2019 Nov 14;14(11):e0225011. doi: 10.1371/journal.pone.0225011 (PMC6855473; doi:10.1371/journal.pone.0225011)
Supplement: S1 Appendix — - Tables A and B. (PDF) [file pone.0225011.s001.pdf]

S1 Appendix. Focus group question and discussion guides

Table A Question and discussion guide for focus groups with surgeons, anaesthetists and pharmacists

Number	Question	Approximate time allocation (mins)
**Q1**	Can you tell us who decides what prophylaxis is given and who administers it? Who is ultimately responsible for this decision?	5
**Q2**	How is this decision for SAP made?	5
**Q3**	Do you routinely check if antibiotics have been given at the right time? Do you check if they are stopped at 24 hours?	5
**Q4**	Current recommendations suggest SAP should be administered within 60 minutes of knife-to-skin. Does this fit in with the general workflow in the theatre?	5
**Q5**	Recent WHO and CDC guidelines now recommend a single dose of SAP for all clean-contaminated procedures. What are your thoughts on this? Will this change practice?	5
**Q6**	Finally, how can SAP prescribing be improved?	5
**Q7**	Any other comments you wish to make?	5

Table B Question and discussion guide for focus groups with nurses

Number	Question	Approximate time allocation (mins)
**Q1**	Can you tell us who decides what prophylaxis is given and who administers it? Who is ultimately responsible for this decision?	5
**Q2**	How is this decision for SAP made?	5
**Q3**	Is there a role for nurses in relation to SAP?	5
**Q4**	Do you routinely check if antibiotics have been given at the right time? Do you check if they are stopped at 24 hours?	5
**Q5**	Current recommendations suggest SAP should be administered within 60 minutes of knife-to-skin. Does this fit in with the general workflow in the theatre?	5
**Q6**	Recent WHO and CDC guidelines now recommend a single dose of SAP for all clean-contaminated procedures. What are your thoughts on this? Will this change practice?	5
**Q7**	Finally, how can SAP prescribing be improved? Should nurses be involved? How can nurses be involved?	5
**Q8**	Any other comments you wish to make?	5
