# Supplementary material for: Influences on surgical antimicrobial prophylaxis decision making by surgical craft groups, anaesthetists, pharmacists and nurses in public and private hospitals
Source: PLoS One. 2019 Nov 14;14(11):e0225011. doi: 10.1371/journal.pone.0225011 (PMC6855473; doi:10.1371/journal.pone.0225011)
Supplement: S2 Appendix — - Table C. (PDF) [file pone.0225011.s002.pdf]

S2 Appendix. Themes and subthemes mapped to the COM-B Model and Theoretical Domains Framework

Table C Themes and subthemes mapped to the COM-B Model and Theoretical Domains Framework

**Themes**	**Sub Themes**	**COM-B Model Components**	**Relevant Domains (Theoretical Domains Framework)**
Low priority for surgical antimicrobial prophylaxis prescribing skills.	Surgical technique of greater importance	Physical Capability	Skills
	Deskilling surgeons		
Prescriber autonomy overrules guideline compliance	Guideline knowledge and awareness of limitations	Psychological Capability	Knowledge
			Memory, attention and decision processes
	Competition as a means to regulate behaviour		Behavioural regulation
Social codes of prescribing reinforce established practices	Hierarchy Rules	Social Opportunity	Social Influences
	Cross-specialty prescriber etiquette		
Need for improved communication, documentation and data for action	Poor documentation and communication	Physical Opportunity	Environmental context and resources
	'Time-Out' supports pre-operative communication; post-operative management is less standardised		
	Data for action		
Fears and perceptions of risk hinder appropriate SAP prescribing.	Fear of infections	Automatic Motivation	Emotion
	Varied risk perceptions across specialties		
	Fear of litigation		
	Risking career progression and job security		
Unclear roles, responsibilities and accountability	The buck stops with the surgeon	Reflective Motivation	Social/ professional role and identity
	Passive prescribing hinders accountability and SAP cessation		Beliefs about Capabilities
	Capacity for role expansion of pharmacists and nurses		Beliefs about Consequences
