# Supplementary material for: Influences on surgical antimicrobial prophylaxis decision making by surgical craft groups, anaesthetists, pharmacists and nurses in public and private hospitals
Source: PLoS One. 2019 Nov 14;14(11):e0225011. doi: 10.1371/journal.pone.0225011 (PMC6855473; doi:10.1371/journal.pone.0225011)
Supplement: Attachment — Submitted filename: Response to Reviewers.docx [file pone.0225011.s003.docx]

**PLOS ONE Minor Revisions Resubmission**

**Response to Reviewers**

27/09/2019

Title of Manuscript: *Influences on surgical antimicrobial prophylaxis decision-making by surgical craft groups, anaesthetists, pharmacists and nurses in public and private hospitals*

Dear Editorial Committee of PLOS ONE,

On behalf of my co-authors, we would like to thank the three reviewers for their kind comments in regards to our manuscript *‘Influences on surgical antimicrobial prophylaxis decision-making by surgical craft groups, anaesthetists, pharmacists and nurses in public and private hospitals’* for possible publication in your PLOS ONE Journal. We were pleased to have received such positive feedback. The following table is a summary of the reviewer and editorial comments and the actions taken to address their concerns. All comments have been acknowledged.

| **Reviewer 1 Comments** | **Authors Response** |
| --- | --- |
| 1. Replace ":" for ";" in lines 341, 528, 551, and 580; | Replaced “;” with “:”. See lines 341, 528, 551, and 580. |
| 2. In line 414/415: check the sequence of tenses; | Reworded. See lines 441-422: *“Pharmacists were noted for their active role in reviewing post-operative SAP when patients returned from theatre”.* |
| 3. In line 481, table 9, column 2, row 5: formatting yielded "communicatio n"; | Column 2 width adjusted to ensure appropriate formatting of words. "communication"; |
| 4. In line 525: insert a space after the full stop: | Space inserted |
| 5. In line 536: hyphenate "time out"; | Hyphenated. See line 536 *“time-out”* |
| 6. In line 554: replace "Australia" for "Australian"; | Reworded sentence to remove saying Australia twice.  New sentence line 553-554.  *“Nurse practitioners are an established profession in Australia, with approximately 1,800 registered in 2019”* |
| 7. In line 555: remove the space before the full stop; | Space removed before full stop |
| 8. In line 571: insert a space after both colons. | Space inserted after both colons. |
| **Reviewer 2 Comments** | **Authors Response** |
| The study gives us insight into barriers to implementing SAP. Authors are to be applauded for their efforts and so do the participants who took a major chunk of time out of their schedules to help understand this issue better. Although it is a snapshot of thoughts in a given geographically limited area, the results do reveal psychological factors that are more universal. | We thank you for your kind comments and agree that even with our limited geographical area, we have identified key psychological factors that are applicable globally. We have already included this issue as a limitation. Please see lines 648-650. |
| Only minor revision I would suggest is to include the participant specific time contributed to the project. if one more vociferous participant contributed more time and opinions than others - this can skew the data and also ultimate take home message due to personal prejudice of this participant. To know each participants total input might help to highlight bias in reporting. | We agree that as a limitation of using focus groups, one cannot guarantee equal representation of each participant.  Unfortunately, we did not transcribe the data to identify different speakers as a means to limit identification and are therefore unable to accommodate Reviewer 2s’ request of calculating each participants’ input per focus group.  We have now included the following statement as a limitation (Lines 696-703):  “A limitation of utilising focus group methodology is the inability to guarantee equal representation of all participants within the group. We acknowledge that some participants are more vociferous than others and therefore their own personal views may project across the focus group. In an attempt to minimise this, the sampling and recruitment of the focus groups was designed to ensure that participants felt comfortable to voice their opinions. If junior staff did not voice their thoughts in the focus group, follow up interviews were planned to be conducted if necessary. An additional focus group solely for surgical registrars and residents was also included to promote participant comfortability”. |
| Many comments seem specialty specific. Can this be reported in a grouped fashion e.g. plastic surgeons vs. colorectal surgeon cohort and their perception of national guidelines etc. This will make it even more meaningful report. | We kindly ask for reviewer 2 to be more explicit in this request. There are many quotes from a range of surgical specialties throughout the manuscript. We are more than happy to accommodate any additional requests reviewer 2 may specifically have regarding this. In our original submission, we were cautious to not overstate our findings and claim one surgical specialty perceived the guidelines to be more helpful than others, as the focus group dialogues that followed from the set questions were dependent on the participants responses. |
| **Journal Requirements** | **Authors Response** |
| 1. Please ensure that your manuscript meets PLOS ONE's style requirements, including those for file naming. The PLOS ONE style templates can be found at [http://www.journals.plos.org/plosone/ s/file](http://www.journals.plos.org/plosone/%20s/file)? id=wjVg/PLOSOne _formatting_sample_main_ body.pdf and [http://www.journals.plos.org/plosone /s/file](http://www.journals.plos.org/plosone%20/s/file)? id=ba62/PLOSOne _formatting_sample_title_authors _affiliations.pdf | Apologies, we have reviewed the linked documents and updated the following: 1. Formatting for headings 1 2 and 3.  2. Ensured sentence case for all headings, note changes to lines 105,109 and 135.  3. Updated the authors affiliations for consistency. Refer to lines 8-21.  4. Supplement data referred to with the correct format. See lines 140 and 165. |
| 2. Please include copies of the focus group discussion guide(s) used in the study, in both the original language and English, as Supporting Information, or include a citation if they have been published previously | Apologies, we have attached the focus groups question and discussion guide(s) as Supplement 1 (Tables 1 and 2) and renumbered our original supplement 1 to be supplement 2 (Table 3). These are referenced in lines 140 and 165 respectively and with separate PDF attachments for S1 and S2 |
| 3.  We note that you have indicated that data from this study are available upon request. PLOS only allows data to be available upon request if there are legal or ethical restrictions on sharing data publicly. For information on unacceptable data access restrictions, please see http://journals.plos.org/plosone/s/data-availability#loc-unacceptable-data-access-restrictions.In your revised cover letter, please address the following prompts:  a) If there are ethical or legal restrictions on sharing a de-identified data set, please explain them in detail (e.g., data contain potentially identifying or sensitive patient information) and who has imposed them (e.g., an ethics committee). Please also provide contact information for a data access committee, ethics committee, or other institutional body to which data requests may be sent.  b) If there are no restrictions, please upload the minimal anonymized data set necessary to replicate your study findings as either Supporting Information files or to a stable, public repository and provide us with the relevant URLs, DOIs, or accession numbers. Please see <http://www.bmj.com/content/> 340/bmj.c181.long for guidelines on how to de-identify and prepare clinical data for publication. For a list of acceptable repositories, please see <http://journals.plos.org/plosone/s/data-availability#loc-recommended-> repositories.  We will update your Data Availability statement on your behalf to reflect the information you provide. | a) Public sharing of our de-identified transcripts was not included in our ethics and local governance applications, nor our consent process. The content within transcripts includes the names of non-participants and workplaces and would allow identification of these non-participants but also may enable identification of participants and their workplaces. For these reasons we cannot share the de-identified transcripts.  This research project was granted ethical approval following low-risk review from the Alfred Hospital Human Research Ethics Committee (HREC) on the 11/10/2017 and the Epworth HealthCare HREC on the 30/10/2017.  Local governance approval was sought from both Alfred Health and Epworth  HealthCare in addition to Melbourne Health (Ethics approval inclusive of Alfred Health HREC approval).  HREC Reference Number: HREC/17/Alfred/143.  Alfred Health local project number: 469-17  Epworth HealthCare local project number: EH2017-270  Melbourne Health local project number: 2017.346  Ethics approval included consent for participation and potential publication.  **CONTACT DETAILS:** 1. Alfred Health Office of Ethics & Research Governance  Email: research@alfred.org.au Phone: +613 9076 3619  2. Epworth HealthCare HREC Coordinator  Email: HREC@epworth.org.au)  Phone: +613 9426 8806 |

We again thank you and the reviewers for your time and for the thorough feedback. We look forward to your response in hopes for approval and publication.

Kind regards,

Ms Courtney Ierano^1,2^- BPharm (Hons), PhD Fellow
National Health and Medical Research Council Centre of Research Excellence: National Centre for Antimicrobial Stewardship (NCAS), Peter Doherty Research Institute for Infection and Immunity Melbourne, Victoria, 3000, Australia
Email: [cierano@student.unimelb.edu.au](mailto:cierano@student.unimelb.edu.au)
Phone: +61424989744

**Co-Author Details:**

Prof Karin Thursky^1,2,3^- MBBS, FRACP, PhD;
Dr Trisha Peel^1,4^- MBBS(Hons), FRACP, PhD.
Dr Arjun Rajkhowa^1,2^- PhD;
A/Prof Caroline Marshall^1,2,5,6^- MBBS, FRACP, PhD;
Dr Darshini Ayton^7^- PhD

**Author Affiliations:**
^1^ National Health and Medical Research Council Centre of Research Excellence: National Centre for Antimicrobial Stewardship (NCAS), Peter Doherty Research Institute for Infection and Immunity Melbourne, Victoria, Australia

^2^ Department of Medicine- Royal Melbourne Hospital, Faculty of Medicine, Dentistry and Health Sciences, University of Melbourne, Parkville, Victoria, Australia

^3^ Department of Infectious Diseases, Peter MacCallum Cancer Centre, Melbourne, Victoria, Australia

^4^ Department of Infectious Diseases, Faculty of Medicine, Nursing and Health Sciences, Alfred Health/Monash University, Melbourne, Victoria, Australia

^5^ Infection Prevention and Surveillance Service, Royal Melbourne Hospital, Parkville, Victoria, Australia

^6^ Victorian Infectious Diseases Service (VIDS), Royal Melbourne Hospital, Parkville, Victoria, Australia

^7^ Department of Epidemiology and Preventive Medicine, Faculty of Medicine, Nursing and Health Sciences, Monash University, Melbourne, Victoria, Australia
